# Diabetes Awareness Campaigns to Prevent Ketoacidosis at the Diagnosis of Type 1 Diabetes: Efficacy on Multiple Outcomes and Predictors of Success: A Systematic Review

**DOI:** 10.3390/jpm14121115

**Published:** 2024-11-21

**Authors:** Elisa Minerba, Evelina Maines, Nadia Quaglia, Ludovica Fedi, Stefania Fanti, Alessandro Fierro, Enza Mozzillo

**Affiliations:** 1 Pediatric Diabetology Unit, Pediatric Department, S.Chiara General Hospital of Trento, Azienda Provinciale per i Servizi Sanitari, Largo Medaglie d’Oro 9, 38122 Trento, Italy; elisa.minerba@studenti.univr.it (E.M.); evelina.maines@apss.tn.it (E.M.); nadia.quaglia@apss.tn.it (N.Q.); stefania.fanti@apss.tn.it (S.F.); 2 Section of Pediatrics, Regional Center of Pediatric Diabetes, Department of Translational Medical Science, Federico II University of Naples, 80138 Naples, Italy; ludoviva.fedi@unina.it (L.F.); al.fierro@studenti.unina.it (A.F.)

**Keywords:** type 1 diabetes, diabetes ketoacidosis, DKA prevention, campaign, youth

## Abstract

**Background/Objectives:** In Italy, the incidence of diabetic ketoacidosis (DKA) at diagnosis of type 1 diabetes (T1D) is still very high (35.7–39.6%), especially in youths. We aimed to determine the efficacy of awareness campaigns to prevent DKA on multiple outcomes and identify success predictors. **Methods:** We searched electronic databases (Pubmed, Cochrane, and Web of Science) for studies published between 1 August 1990 and 1 August 2024. The review included studies that focused on children under 18 years old, and outcomes were measured by comparing before and after implementing the campaigns in the same area and between areas where interventions took place or not. **Results:** Of 236 records identified, 15 were eligible for analysis. After campaign implementation, the pooled DKA reduction resulted between 1% and 65.5%, based on the characteristics of the campaigns. A decrease in the rate of acute complications, such as cerebral edema, was reported. Hemoglobin A1c (HbA1c) at onset showed a mean reduction of 0.7–5.1%; C-peptide increased in patients without DKA at diagnosis, and length of hospitalization decreased. Campaign costs were lower than the costs of treating subjects with DKA. **Conclusions:** This review demonstrated that DKA awareness campaigns effectively reduce DKA incidence and improve other parameters, such as acute complications, HbA1c and C-peptide levels, length of hospitalization, and costs, among youths with T1D. To be effective, campaigns must follow specific principles of target population, modality, and minimal duration, as reported in this review.

## 1. Introduction

Diabetic ketoacidosis (DKA) is a severe acute complication of type 1 diabetes (T1D) that is associated with increased mortality, morbidity, and hospitalization costs [1]. It can occur at the clinical diagnosis of T1D in children and adolescents and predicts poor long-term metabolic control [1].

In new-onset T1D, DKA often results from a delay in diagnosis. The frequency of DKA at T1D onset varies from 12.8% to 80% worldwide [2]; in Italy, the frequency of DKA at the onset of T1D is still very high (35.7–39.6%), especially in the younger age group [3], and, in our region, Trentino Alto Adige, has been on average 46% in the last decade [4]. The frequency of DKA and severe DKA increased during the early pandemic and continued throughout 2020 [3,5,6,7].

The frequency of DKA at T1D onset is lower in countries where the background incidence of T1D is higher [8], and the prevalence of DKA is still high due to the lack of awareness about symptoms of T1D among parents and healthcare providers [9]. A recent systematic review by Rugg-Gunn et al. [10] demonstrated that a family history of T1D, antibodies screening programs, and a higher level of parental education are all factors associated with decreased risk of DKA at T1D onset. All these factors are connected with raising awareness of the most common presenting symptoms [11].

In recent years, the increasing incidence of T1D globally has pushed different countries, including Europe and the United States, to implement antibody screening programs [12]. The main objective of T1D screening programs is to identify individuals at risk or in an early stage to avoid DKA and offer them interventions to prevent or postpone the disease. Over time, the goal will be to integrate such screening into the standard care of national and regional health systems. On 17 September 2023, the Italian Parliament approved a law introducing nationwide screening for T1D in the general population aged 1–17 as part of the public health program to reduce the effects of this chronic condition. However, annual islet autoantibody (IAb) screening from early childhood to early adolescence [12] or at landmark ages (2, 6, and 10 years), as suggested by other authors [13,14,15], is cost-saving only if it reduces the rate of DKA by 20%, with a subsequent lower hemoglobin A1c (HbA1c) by 0.1% over a lifetime [16].

An alternative or complementary strategy consists of implementing public educational campaigns, and the Parma campaign in 1991 demonstrated the effectiveness of a public education program in reducing the frequency of DKA at diabetes diagnosis in a restricted area in Italy [17]. Subsequent analysis suggested that such prevention campaigns should be repeated periodically to maintain effectiveness over time [18]. However, what makes the campaign effective is an area of inquiry [17], and information on the target population, modality of the intervention, and how to assess impact are a matter of debate. A recent systematic review and meta-analysis by Cherubini et al. [3] demonstrated that DKA awareness campaigns effectively reduce DKA among children and adolescents with T1D, and the authors considered only the frequency of DKA as an outcome.

Therefore, we performed a systematic review on the efficacy of multiple outcomes (comprehensive of HbA1c, C-peptide, complications, hospitalization duration, and costs) and possible predictors of the success of prevention campaigns.

## 2. Search Strategy

We searched electronic databases (PubMed, Cochrane, and Web of Science) for studies published between 1 August 1990 and 1 August 2024. The search terms or “MeSH terms” (Medical Subject Headings) for this review included different combinations: “type 1 diabetes” or “T1D” AND “campaigns” or “awareness” AND “ketoacidosis.”

Inclusion criteria were (1) studies on youths (0–18 years) with T1D; (2) randomized clinical trials and observational studies; (3) at least 100 participants (as in a previous review, studies with at least this sample size were able to give significant outcome differences) [3]; (4) describing the characteristics and the impact of the campaign; (5) full-text availability. 

We excluded case reports, studies with less than 100 pediatric participants, and studies that did not fulfill inclusion criteria. Languages other than English were not a priori exclusion criteria. 

Two independent investigators (EMI and SF) screened for inclusion in the title and abstract of all the studies identified using the search strategy. Any discrepancies between them were resolved by consensus or consultation with a third investigator (LF). After abstract selection, four investigators conducted the full paper analysis (EMA, NQ, AF, EMO). 

For each study, in the full paper, we evaluated the reference details, the population and study characteristics, the methodology used (public posters or flyers, lectures, social networking, and free phone line), the outcomes measured, and the results. These data were reported in Table 1, and the main findings in Table 2. 

Two authors (EMO, EMA) independently assessed the certainty of the evidence for each of the outcomes. Risk of bias in the study design, imprecision of estimates, inconsistency across studies, indirectness of the evidence, and publication bias are reported during the study presentation in the results section. A meta-analysis was not conducted due to the heterogeneity in the design of the campaigns in terms of target population, duration, intervention modality, and outcome measure. This review was not registered in PROSPERO. All the databases used for the analysis are available for consultation under request.

## 3. Results

In total, 340 studies were found by using the MESH defined above. After removing duplicates, 263 studies were analyzed. We reviewed titles and abstracts, and 241 additional records were excluded: 22 review articles, 36 including only participants older than 18 years, 1 study with a number of subjects less than 100, 168 studies reporting an outcome different from that of interest, and 14 for the study design. A total of 16 full-text manuscripts were assessed for eligibility: after full-text examination, 1 study was excluded, and a final number of 15 studies was included in this review.

**Figure 1:**
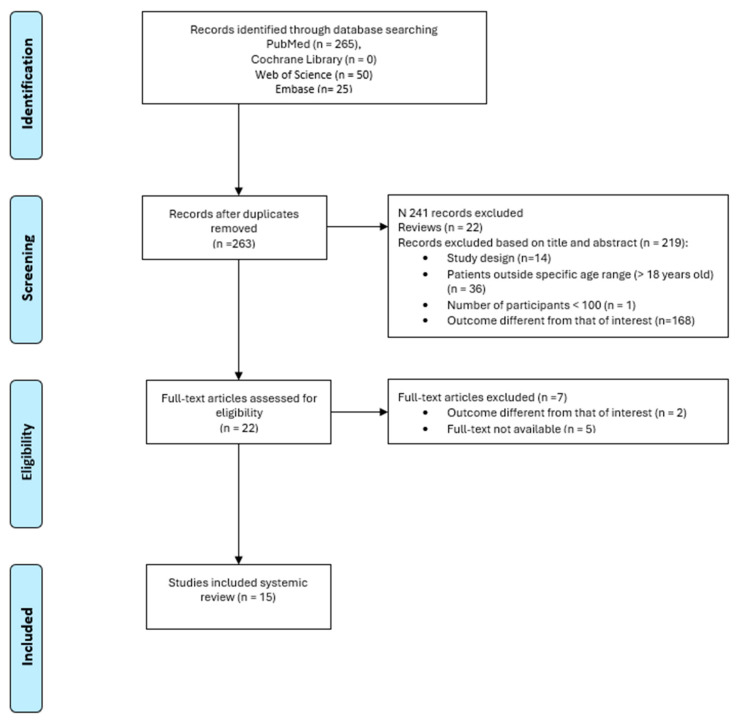
Publication selection process summarized by the PRISMA flowchart.

The PRISMA flow diagram (Figure 1) summarizes the publications screening process.

All the studies were prospective longitudinal in design. The number of patients enrolled in the studies was between 106 and 438,232 [17,27]. In Table 1, we summarize the characteristics of the studies included in the review (target population, geographic area, description of intervention, campaign duration, results). Below, we present the main results divided into five categories: frequency of DKA at the onset, acute complications, HbA1c, C-peptide, duration of hospitalization, and costs. The summary of evidence for each outcome is reported in Table 2.

### 3.1. Frequency of DKA

Overall, 14 studies included the frequency of DKA at diagnosis in their results. Four studies evaluated the effectiveness of interventions by comparing the frequency of DKA in an area where the preventive intervention was implemented and in a control area where no campaign was conducted. The other ten studies compared the frequency of DKA in the same area before and after the prevention campaign. The pooled reduction in DKA was between 1% and 65.5%. The DKA incidence after the campaign ranged from 5.9% to 47.6%, proving the efficacy of interventions in some studies but not in others.

Analyzing effective campaigns, the study of Cangelosi et al. [26] measured a DKA frequency of less than 6% after the 5-year duration of the campaign, reporting a decreased frequency in the intervention and control areas. The decrease in DKA frequency in the control area was hypothesized to be associated with a not-programmed sensibilization promoted by pediatricians, the parents’ community of children with T1D, or new graduates in Medicine and Pediatrics at the University of Parma who were informed of the prevention program in the area of Parma. Other studies [17,18,26] showed a significant decrease in DKA incidence, with 25% or less frequency of DKA.

Instead, the studies of Derraik et al. [28], Rabbone et al. [29], Fritsch et al. [22], Lansdown et al. [21], and Choleau et al. [24] reported an increase or a nonsignificant decrease in the frequency of DKA after implementing the interventions. Derraik et al. [28] planned a campaign that lasted only a year and targeted the general population, not a specific one; moreover, the natural fluctuation in DKA incidence from year to year and other factors may influence the result of their study. In addition, more than 40% of all those diagnosed with T1D over the study period were non-European children, indicating that the campaign design did not manage to reach the more vulnerable populations. Rabbone et al. [29] measured an increased DKA frequency after the interventions, probably related to the limited duration of the study (only two years) and the shortage of family pediatricians observed in Italy in those years. Moreover, the study did not include either a control group or a method to verify that the posters were displayed and seen by the target population. The survey of Fritsch et al. [22] measured a non-significant decrease in the rate of DKA incidence. Their 1-year campaign involved displaying posters and flyers and educational sessions, but the dissemination of the interventions was covered by general practitioners and not by primary pediatric care. Another study that found a non-statistically significant decrease in the rate of DKA was the one of Lansdown et al. [21]: in this study, the questionnaire survey showed that only a few parents and GPs had seen the posters and were aware of the media coverage.

### 3.2. Complications

Six studies included acute complications in the results, comparing the rate of complications before and after the campaign or in patients with or without DKA at the onset. Uçar et al. [23] and Patwardhan et al. [27] found a reduction in the severity of DKA. Rabbone et al. [29] described a nonsignificant (*p* = 0.548) decrease in the rate of cerebral edema in patients with DKA after vs. before the campaign, while Patwardhan et al. [27] reported an increase in this outcome, even though this result was classified as non-statistically significant because of the restricted number of participants and the sporadic nature of this complication. The interventions carried out by Ahmed et al. [25] determined an absence of severe complications during the 4-year campaign, and the overall morbidity and mortality in the DKA cohort were comparable to those of other population-based studies.

### 3.3. HbA1c

Seven studies measured HbA1c as an outcome of the effectiveness of the prevention campaign at the diagnosis and during the follow-up [17,23,25,27] and/or compared the value in the patients with vs. without DKA at diabetes diagnosis [23,26,27,28,30]. In each of these studies, the group of patients without DKA at the onset had a lower value of HbA1c than the ones with DKA, with a reduction in its rate from 0.7% to 5.1% and a range from 9.1 to 11.8%.

One of the best results was achieved by Cangelosi et al. [26], who reported a decrease in HbA1c from 13.7% to 9.9% in patients without DKA at diagnosis of T1D. They attributed this result to the awareness raised by the campaign on early symptoms of T1D at onset, leading to a shorter period of relative insulin deficiency status in children. Ahmed et al. [25] and Uçar et al. [23] reported lower HbA1c values at diabetes diagnosis after the campaign, confirming the interventions’ effectiveness. The study of Vanelli et al. [17] compared HbA1c in children from the Parma area with others from two nearby areas, showing a decrease in the ones coming from the province of Parma, where the prevention campaign was implemented.

### 3.4. C-Peptide

Only two studies included C-peptide as an outcome and analyzed its relation with DKA. In the study of Vanelli et al. [17], there was an increase in the C-peptide value of 0.03 ng/mL (from 0.12 to 0.15 ng/mL in children from Parma vs. from two nearby areas). Uçar et al. [23] associated the significant increase in c-peptide of 0.10 ng/mL (from 0.50 to 0.60 ng/mL) with the absence of DKA at the disease onset because of the limited destruction of beta-cells.

### 3.5. Length of Hospitalization

Three studies measured the length of hospitalization. The study of Derraik et al. [28] reported an association between DKA and hospitalization, showing that children without DKA at T1D diagnosis were hospitalized for fewer days (5.5 vs. 4.7 days in children with DKA vs. without it, *p* = 0.036). Darmonkow et al. [31] and Vanelli et al. [17] analyzed the length of hospitalization before and after the campaign, and a reduction in the hospitalization duration was reported after implementing the interventions. In the study of Vanelli et al. [15], there was a difference of 8 days of hospitalization in the children from the area of Parma compared to the two nearby Provinces. Darmonkow et al. [31] found a significant difference in the length of hospitalization comparing data before, during, and after the campaign (45.8%, 30.9%, and 40.6%, respectively, with *p* < 0.001).

### 3.6. Costs

Three studies reported the costs of the campaign. They estimated the difference between treatment costs in patients with DKA diagnosis and those without it. Each study calculated an increase in hospitalization costs associated with DKA, with a range from a minimum of USD 12,193 in the study of Derraik et al. [28] to a maximum of USD 141,101 in the one of Vanelli et al. [17]. Jelley et al. [19] reported an estimated cost savings of USD 85,000 for DKA-related hospitalizations. Moreover, Vanelli et al. [18] and Jelley et al. [19] included the total cost of the campaign and considered the campaign cost-effective or even costless, given the benefits obtained.

## 4. Discussion

This review aimed to determine the efficacy of diabetes awareness campaigns on DKA frequency at T1D onset, HbA1c, complications, C-peptide, length of hospitalization, and costs. We conducted accurate research in scientific literature to identify all the studies related to the topic. Here, we discuss the results of the various campaigns to draw some conclusions based on the evidence from the literature.

Diabetes awareness campaigns effectively reduced the frequency of DKA at diagnosis in children, but the results were very different along with the studies, with an impact ranging from −1% to −65%. Campaigns were more successful in reducing DKA frequency when they included the following elements:

1. Target population: most campaigns addressed three specific target populations, often including families with young children, schoolteachers, and healthcare professionals [21,22,28]. Campaigns should inform children’s parents and school teachers about the symptoms of T1D: polyuria/polydipsia and enuresis were the main alerting symptoms on which the Italian campaign was based [18]. Health professionals have to rapidly intervene when parents report symptoms; therefore, the targeted message is that the quicker the diagnosis is, the less frequent the DKA, particularly in younger children. A simple search for glucose in urine or measurement of blood glucose using test strips is more than enough to refer a child to the closest emergency room [24].
  Prevention campaigns carried out in a delimited geographical area (such as a Region of Italy) give better results than a national area such as Italy or Austria [18];
2. Multiple warning tools have to be provided:
  (i) A system to evaluate that the target population has seen and read the posters and flyers with information about DKA and diabetes diagnosis [17,19,20,21,22,23,24,25,26,27,31], as this has been a limit in previous studies that had no way to verify if all the posters have been displayed in the ambulatory setting or the schools [29];
  (ii) Targeted lectures or educational meetings [20,22,25,26,27,31];
  (iii) The equipment to measure blood glucose levels and check for the presence of glycosuria, if given to family doctors, improved campaign efficacy [17,20];
  (iv) Posts on social media and announcements on television or radio are innovative communication tools to be used for awareness campaigns [19,21,22,24,26,29];
  (v) A toll-free telephone line directly connected to the diabetes team of the department [17,18,26].
3. Campaign duration: the campaign should last at least 2 years [22,28,29] and must be renewed every five years [18] to maintain its effectiveness; the Parma Campaign was still effective eight years after the campaign was promoted [18].
4. A monitoring system of the campaign’s effectiveness by interviewing the target population and estimating the trend of DKA are fundamental to adjusting and updating the campaign. An acceptable target for the frequency of DKA has been suggested to be less than 5%, as observed in screening programs based on the islet autoantibodies test [3].

Our findings regarding DKA frequency are similar to the ones reported by Cherubini et al. [3] in 2021 in a systematic review, as they reported a significant reduction in DKA frequency at T1D diagnosis. Some characteristics of the campaigns emerged more frequently as favorable: a small intervention area with less than 700,000 inhabitants, a well-defined target population, and multiple warning tools. We also analyzed other outcomes, and, as expected, the impact of the campaigns was positive in reducing the severity of DKA [23,27] and acute neurological complications associated with DKA, such as loss of consciousness, coma, cerebral edema, and mortality [25,29].

A decrease in the rate of HbA1c at diagnosis of T1D (from −0.7% to −5.1% reduction) was detected in all the studies that analyzed this outcome [17,23,25,26,27,28,30], as well as a higher c-peptide reserve was reported, concordant with preservation of more functional residual beta-cells [17,23,32]. Moreover, the reduced frequency of DKA at the T1D onset led to better glycemic control during follow-up, as evidenced in terms of HbA1c [33], and this could decrease the risk of long-term complications, including brain damage associated with hyperglycemia and hypoglycemia, as well as vascular complications [34].

Due to the reduction in DKA frequency, the length of hospitalization was reduced in subjects without DKA at the disease onset after the awareness campaign [17,31]. Therefore, by reducing the DKA frequency and days of hospitalization, the campaigns reduced the costs related to DKA treatment and possible hospital-acquired infections. In the long term, additional economic benefits are associated with reducing chronic complications due to the better metabolic control of patients who did not have DKA at diabetes onset. Conversely, the cost of a prevention campaign has been reported as less expensive [18,19].

The strengths of this systematic review are as follows:

1. It is the first one presenting the analysis of other outcomes besides DKA frequency, on which a recent systematic review already exists; as a matter of fact, this review also includes data on HbA1c at onset, complications linked to DKA, C-peptide, length of hospitalization and costs;
2. It includes studies from 1990 to analyze every form and modality of prevention campaign published nowadays;
3. We were able to report the characteristics that give efficacy to this type of campaign.

The limitations of this study are as follows:

1. We did not report a metanalysis about the DKA frequency because it was already included in the previous review of 2021 [3].
2. It was impossible to create a metanalysis about other parameters (for example, HbA1c at onset) considering the heterogeneity of the data in terms of the size and type of the target population, study design, campaign duration, and multiple implementation modalities. In particular, the duration of the campaign was quite different among studies.

For clinical practice, results from this review give us the principles and modalities to design a diabetes awareness campaign in our region, which we expect to drop the frequency of DKA, reduce costs, and improve metabolic control in subjects with T1D. 

For research, we planned to evaluate prospectively whether implementing a prevention campaign will improve HbA1c during T1D diagnosis and during follow-up, as well as Time in Range and other glucose metrics.

## 5. Conclusions

The frequency of ketoacidosis at diabetes onset is still high in Italy because of the delay in diagnosis due to a lack of awareness among parents, other caregivers, schoolteachers, or healthcare professionals about the symptoms of T1D in children [3]. Antibodies screening of the general population could help reduce DKA frequency at disease onset and allow participation in trials with c-peptide-preserving drugs [35]. At the same time, diabetes awareness campaigns are an effective tool in lowering DKA and costs and improving metabolic control in the future. However, these preventive interventions must be organized by following specific principles of target population, modality, and minimal campaign duration, which we reported above.

## Figures and Tables

**Table 1:** Summary of the findings of the 15 studies included in this review.

Study (Author, Year)	Region	Children with T1D Onset Age and Number; Population Target of the Campaign	Description of Intervention	Duration	Results				
					Frequency of DKA	Complications	HbA1c	C-Peptide	Hospitalization and Costs
Vanelli et al., 1999 [17]	Italy, Parma	6–14 years 438,232 Students, family with young children, schoolteachers, primary care physicians	Educational session of 1-h duration to general pediatricians. Posters about medical information of DKA. Postcards with criteria for the diagnosis of T1D according to the WHO. Devices for measurement of capillary blood glucose and glycosuria Toll-free information phone number.	8 years (1991–1998)	Decrease in rate of DKA from 78% to 12.5% (*p* < 0.0001) in the province of Parma vs control provinces. Decreased frequency of DKA from 83% to 12.5% in children from Parma vs. from two nearby areas.	-	Decreased rate of HbA1c from 14.5% to 9.4% in children from control provinces vs from Parma.	Increased level of C-peptide C from 0.12 to 0.15 ng/mL in children from Parma vs. from two nearby areas.	Reduction in length of hospitalization from 13.3 days to 5.4 days in patients from Parma vs. from Reggio Emilia and Piacenza. The total cost of the campaign was USD 23,470. Lower cost from treatment and education from USD 196,457 to USD 53,356 in patients without DKA vs. with DKA.
Vanelli et al., 2008 [18]	Italy, Parma	6–14 years not mentioned Students, family with young children, schoolteachers, general pediatrician	Poster indicating early symptoms of T1D, flyers with guidelines about T1D diagnosis.	8 years (1998–2006)	Reduction in DKA frequency from 27% to 19% (*p* < 0.0001) in the province of Parma vs. control provinces.	-	-	-	The total cost of the campaign was $23,470.
Jelley et al., 2010 [19]	USA, Oklahoma	Not mentioned 193 Students, schoolteachers, primary care physicians	Informational posters, postcards indicating early symptoms, media blitz on local television, advertising on a regional newspaper.	6 months (2009)	Decreased rate of DKA from 29.9% to 23% in the year of the campaign vs. 6 months before it (*p* < 0.05).	-	-	-	The total cost of the campaign was USD 1100.
King et al., 2012 [20]	Australia, Gosford	0–18 years 328 Schoolteachers, primary care physicians.	Posters and postcards. Educational session. Glucose and ketone testing equipment. A toll-free diabetes information phone number.	2 years (2008–2010)	Reduction in DKA frequency from 37.5% to 13.8% (*p* < 0.03). Any change in the control regions.	-	-	-	-
Lansdown et al. 2012 [21]	UK, Wales	<15 years 3033 Schoolteachers, primary care physicians	Advertising posters. Television and radio interviews about symptoms and late diagnosis.	19 years (1991–2009)	Non statistically significant reduction in DKA frequency from 27.5% to 25.6% (*p* < 0.72).	-	-	-	-
Fritsch et al., 2013 [22]	Austria, Vienna	<15 years 4038 General population, kindergartens, primary and secondary schools, pharmacies, primary care physicians.	Posters focused on early symptoms of DKA. Medical journals about DKA. Educational sessions about diabetes and DKA. Broadcast in tv and articles on regional newspaper.	22 years (1989–2011)	Non-significant reduction in DKA frequency from 37.8% to 36.8% (*p* > 0.05).	-	-	-	-
Uçar et al., 2013 [23]	Turkey, Istanbul	8–5 years (0.5–17.5 years) 401 General population, family, students, schoolteachers.	Awareness posters and brochure on diabetes. Educational material on specific websites.	2 years (2011–2012)	Decreased DKA frequency from 49.3% to 23.9% (*p* < 0.001) in patients of 2011–2012 vs. the ones of 2003–2010	-	-	-	-
Choleau et al., 2014 [24]	France	<15 years 1299 General population, schoolteachers, primary care physicians.	National information campaign. Publication on general and specific newspapers. Posters. Interviews and spots on national and regional televisions and radios.	1 year (2009)	Decreased frequency of DKA from 43.9% to 40.5% after 1 year (*p* = 0.08).	In DKA vs. not DKA: loss of consciousness 30.2% vs. 0.30%, deep coma 3.2% vs. 0 and of hospitalization in Intensive Care Units 53.6% vs. 5.3%	-	-	-
Ahmed et al., 2015 [25]	North of Saudi Arabia	<12 years 541 General population, family, schoolteachers, primary care physicians.	Educational interventions and sessions. Poster and flyers. Media coverage.	4 years (2010–2014)	Reduction in DKA frequency from 48% to 39% (*p <* 0.01)	Any severe complications during the intervention years. Rates of mortality and morbidity of DKA cohort were comparable to other population-based studies.	Decreased rate of HbA1c from 10.0% to 9.1% after the campaign (*p* < 0.001)	-	-
Cangelosi et al., 2017 [26]	Italy, Parma	6–14 years 135 General population, schoolteachers, primary care physicians.	Poster and leaflets. Local radio announcements.	5 years (2012–2016)	Decreased rate of DKA frequency from 52.7% to 5.9% in children from province of Parma vs. from two other nearby provinces (*p* = 0.002).	No cases of severe DKA.	Decreased rate of HbA1c from 13.6% to 9.9% in patients without DKA vs. with DKA.	-	-
Patwardhan et al., 2018 [27]	Australia, Queensland	Age: 0–18 years 106 Health professionals	Educational section of 1-h duration.	1 year (2015–2016)	Reduction in rate of DKA frequency from 54.9% to 25% (*p* = 0.01).	Decreased rate of severe and moderate DKA and a decrease in patients being admitted to the ICU for DKA at first presentation. Increase in rate of cerebral oedema from 0.5–0.9% to 1.96% in median vs. in patients with DKA during this study.	Reduction in rate of HbA1c from 12.74% to 11.45% in patients without DKA vs. with DKA (*p* = 0.09).	-	-
Derraik et al., 2018 [28]	New Zealand. Auckland	<16 years 460,000 General population, primary care physicians	Posters delivered to mailboxes of individual residential households and to general practitioners and surgery staff to be displayed in waiting rooms.	2 years (2015–2017)	Increase in rate of DKA frequency from 27% to 28.8%.	-	in rate of HbA1c from 12.5% to 11.8% in patients without DKA vs. with DKA (*p* = 0.28).	-	The total cost of the campaign was USD 40,107. This campaign prevented three cases of DKA, which means it saved approximately from USD 13,369 to USD 33,569 per case. Decreased in rate of hospitalization from 5.5 days to 4.7 days in not DKA cohorte vs. DKA cohorte.
Rabbone et al., 2020 [29]	Italy	0–18 years Number: 2361 children and 250 schools	A national awareness campaign. Poster and bimonthly magazine with information regarding diabetes symptoms. Short commercial on tv and announcements on national and regional television channels.	2 years (2015–2017)	Increase in rate of DKA frequency from 38.5% to 47.6% (*p* = 0.002).	Decreased rate of cerebral oedema from 0.53% to 0.35% after intervention.	-	-	-
Holder & Ehehalt, 2020 [30]	Germany, Stuttgart	0–18 years with median age 4.5 years 44,000 General population, schoolteachers, family, students, pharmacists, pediatrician	Lecture to schoolteachers Public posters, flyers, newsletters illustrating typical clinical symptoms of T1D.	3 years (2015–2017)	Decreased DKA frequency from 28.3% to 16.1% in patients of 2015–2017 vs. 2011–2013 (*p* < 0.02).	-	Decreased HbA1c of 1.4% in patients without DKA vs. with DKA (*p* < 0.0001)	-	-
Darmonkow et al., 2021 [31]	Canada, Québec	<25 years 232 General population, family, students, schoolteachers, primary care physicians, pharmacists.	Educational sections, posters, and a DKA prevention kit.	6 years: three periods of 2 years each (2009–2010, 2011–2012, 2013–2014)	-	-	-	-	Decrease in hospitalization for DKA from 45.8% to 40.6% after vs. before the campaign in children from 0 to 19 years old.

**Table 2:** Summary of the evidence for each outcome.

Title, Authors and Publication Year	Number of Children and Duration of Follow-Up	Frequency and Decrease in DKA	HbA1c (in Patients without DKA)	C-Peptide	Hospitalization	Costs of Patients with DKA/without DKA
Vanelli et al., 1999 [17]	438,232; 8 years	−12.5 (−65.5)	9.4 (−5.1) Parma vs. Control cohort	0.15 (+0.03)	3 days less (Parma vs. Control cohort)	+ EUR 131,090
Vanelli et al., 2008 [18]	-; 8 years	19 (8)	-	-	-	-
Jelley et al., 2010 [19]	193; 6 months	23 (−6.9)	-	-	-	+ EUR 75,841
King et al., 2012 [20]	-; 2 years	13.8 (−23.7)	-	-	-	-
Lansdown et al., 2012 [21]	3033;19 years	25.6 (−1.9)	-	-	-	-
Fritsch et al., 2013 [22]	4038; 22 years	36.8 (−1.0)	-	-	-	-
Uçar et al., 2013 [23]	401; 2 years	23.9 (−25.4)	10.3 (−0.7)	0.60 (+0.10)	-	-
Choleau et al., 2014 [24]	1299; 2 years	40.5 (−3.4)	-	-	-	-
Ahmed et al., 2015 [25]	541; 4 years	39.0 (−9.0)	9.1 (−0.9) after vs. before the campaign	-	-	-
Cangelosi et al., 2017 [26]	135; 5 years	5.9 (−46.8)	9.9 (−3.7)	-	-	-
Patwardhan et al., 2018 [27]	106; 1 year	25.0 (−29.9)	11.5 (−1.3)	-	-	-
Derraik et al., 2018 [28]	-; 2 years	28.8 (+1.8)	11.8 (−0.7)	-	1 day less (in patients without DKA vs. with DKA)	+(EUR 10,062–30,188) 2.2 times higher
Rabbone et al., 2020 [29]	2361; 2 years	47.6 (+9.1)	-	-	-	-
Holder & Ehehalt, 2020 [30]	44,000; 3 years	16.1 (−12.2)	(−1.4)	-	-	-
Darmonkow et al., 2021 [31]	232; 6 years	-	-	-	5.2% less (0–19 years old) after the campaign	-

## Data Availability

All databases generated for this study are included in this article.
